# Modification of the Properties of Polymer Composites in a Constant Magnetic Field Environment

**DOI:** 10.3390/ma14143806

**Published:** 2021-07-07

**Authors:** Ewa Miękoś, Michał Cichomski, Marek Zieliński, Tomasz Klepka, Dariusz Sroczyński, Anna Fenyk

**Affiliations:** 1 Department of Inorganic and Analytical Chemistry, Faculty of Chemistry, University of Lodz, Tamka 12, 91-403 Lodz, Poland; marek.zielinski@chemia.uni.lodz.pl (M.Z.); dariusz.sroczynski@chemia.uni.lodz.pl (D.S.); anna.fenyk@chemia.uni.lodz.pl (A.F.); 2 Department of Materials Technology and Chemistry, Faculty of Chemistry, University of Lodz, Pomorska 163, 90-236 Lodz, Poland; michal.cichomski@chemia.uni.lodz.pl; 3 Department of Technology and Polymer Processing, Faculty of Mechanical Engineering, Lublin University of Technology, Nadbystrzycka Street 36, 20-618 Lublin, Poland; t.klepka@pollub.pl

**Keywords:** polymers, biopolymers, composites, constant magnetic field (CMF), magnetohydrodynamics

## Abstract

In this paper, polymer composites based on polylactide (PLA) and epoxy resin (Epidian 5) were studied in terms of the influence of magnetic induction on their changes in physicochemical properties. The composites contained admixtures in the form of magnetite (Fe_3_O_4_) and crystalline cellulose (Avicel PH-1010) in the amount of 10%, 20%, and 30% by weight and starch in the amount of 10%. The admixtures of cellulose and starch were intended to result in the composites becoming biodegradable biopolymers to some extent. Changes in physical and chemical properties due to the impact of a constant magnetic field with a magnetic induction value B = 0.5 T were observed. The changes were observed during tests of tensile strength, bending, impact strength, water absorbency, frost resistance, chemical resistance to acids and bases, as well as through SEM microscopy and with studies of the composition of the composites that use the EDS method and of their structure with the XRD method. Based on the obtained results, it was found that the magnetic induction value changes the properties of composites. This therefore acts as one method of receiving new alternative materials, the degradation of which in the environment would take far less time.

## 1. Introduction

The increasing demand for plastic products has contributed to a sharp increase in the amount of landfilled polymer waste. These plastics bring many benefits to society, for example, an increase in the product lifespan or reduction of CO_2_ emissions into the atmosphere [1]. However, problems with their long-term biodegradation process have caused them to remain in all elements of the natural environment for years. It is estimated that over 8 million tons of plastic end up in the oceans every year [2]. It is currently found in all major ocean currents, polar seas, and deep-sea sediments in a wide range of particle sizes [3]. Therefore, the interest in the search for new alternative materials has increased in the recent years. The addition of natural admixtures not only endows them with new properties, but also facilitates their degradation in the environment. “Green chemistry” refers to a sustainable environment using biocompatibility, biodegradability, as well as economical and simple materials based on a variety of biopolymer matrices, e.g., chitosan, starch, cellulose, gelatin, alginate, polyhydroxyalkanoate, carrageen, etc. They are widely used in the following areas: organic food, packaging, special biomedical dressings, and water treatment technologies [4]. It was also proven that the properties of polymers and composites can be influenced by the use of a constant magnetic field during their production, especially when using magnetic admixtures. Examples of such materials are composite films made of graphene nanoflakes and Fe_3_O_4_ magnetite nanoparticles. They were obtained in the presence of a constant magnetic field and, for comparison, without its participation. Polystyrene sulfonic acid, i.e., PEDOT, was used as a stabilizing polymer matrix. The morphology of the film depended strongly on the presence of the magnetic substance and the constant magnetic field. Its structure became more porous in a magnetic field than in its absence. The layers obtained without a magnetic field have an activation character of conductivity, whereas the thin layers formed in the magnetic field have metallic conductivity [5,6,7,8,9].

Another example of scientific interest in composites containing magnetic particles in the polymer matrix is agar, a biocompatible polymer that is a matrix for magnetic particles of iron carbonyl. Their presence makes the polymer capable of reacting to an external magnetic or electromagnetic field. Using photothermal radiometry (PTR) in the backpropagation, the heat transfer properties of composites containing different concentrations (5, 10, 15, 20, 25, and 30% *w/w*) of iron carbonyl particles were examined. The morphology of iron carbonyl-agar composites in a magnetic field was also assessed using scanning electron microscopy (SEM). The results showed a dominant effect of iron carbonyl concentration on the degree of particle ordering induced by the magnetic field, which is consistent with the behavior of the thermal diffusivity and thermal conductivity. Agar served as an excellent matrix for composites with an admixture of iron carbonyl particles. At 20% carbonyl concentration, the magnetic field-ordered composite can be considered a promising “smart” material for the treatment of hyperthermia in the field of biomedicine [10,11,12,13]. Scientific research in the field of polymers is the driving force behind the progress of civilization. Furthermore, at present, research into the synthesis of new polymeric materials provides increasingly sophisticated solutions for virtually all fields of technology and economy. Most of the directions of that research concern so-called smart polymeric materials with the basic functions of discerning changes processing the obtained information, and responding to these changes. Smart materials are capable of responding to external stimuli, e.g., in the magnetic field, by significantly changing their properties in order to obtain the desired and effective response to these stimuli. Such materials are currently used in almost every field of science or technology. Magnetic particles contained in the polymer, in order to achieve large magnetic moments, position in the direction of the external magnetic field. Intermolecular forces cause the particles to attract one another, leading to their aggregation in complex networks, shortening the distance between them, and thus increasing the material rigidity. Mechanical properties can be modulated by magnetic field. Polymer composites gain also new and improved physical and chemical properties. Their water absorption capacity is reduced and their chemical resistance to acids and alkali as well as frost resistance is increased. Thanks to their unique properties, such materials can find wide application in the space industry, electrical engineering, or automotive industry. In addition, magnetorheological abrasive polishing of complexly shaped objects with application of a constant magnetic field is one of the most promising methods of surface treatment of machine components. This method can be applied to objects of complex shapes using abrasive masses based on polymers and abrasive grains with ferromagnetic properties. Magnetically mixed soft FeSiCr composites (amorphous FeSiCr powder of 16.7 μm particle size) with the addition of various carbonyl iron powder (CIP) content of an average particle size of 4.75 μm were also studied. The effect of iron carbonyl on the microstructure, density, and magnetic properties of amorphous FeSiCr was analyzed. When the CIP content increased from 0% to 50% by weight, the relative density increased from 75.9% to 84.9% and the magnetic permeability of the sample heat-treated at 500 °C rose from 19.1% to 36.8%, i.e., by 92.7%. As the iron carbonyl content increased, the rate of increase in the composite density decreased gradually [14,15,16,17,18,19].

Another example of the effect of magnetic and natural particles added on the properties of materials are composites consisting of a biopolymer chitosan matrix and a hybrid CoFe_2_O_4_-cellulose filler. The introduction of cellulose into the oxide-chitosan composite significantly modifies its magnetic and mechanical properties. The presence of filler in the chitosan matrix hindered the movement of molecules, which resulted in a decrease in activation energy. The addition of cellulose to the filler increased the coercive field H_c_ compared to pure CoFe_2_O_4_ powder from 0.1453 to 0.2033 T. The introduction of the filler resulted in an improvement in Young’s modulus and tensile strength compared to chitosan without filler. For nanocellulose filler composites, this strength was more than twice that of pure chitosan [20,21,22].

The effect of a constant magnetic field on the ordering of cellulose nanocrystals (CNCs) in the starch matrix and its effects on the physical, chemical, and mechanical properties of nanocomposites were also studied. Two types of nanocrystals—plant and tunicate—were studied. Two different kinds of CNC, i.e., plant-CNC and tunicate-CNC and its hybrid combination, are studied to understand the effect of the aspect ratio of CNC on the properties of the nanocomposite. Nanocomposites with tunicate sourced CNC showed higher tensile strength and modulus, and lower water vapor permeability compared to plant sourced CNC. These properties are higher for nanocomposites prepared under a constant magnetic field. The modulus of starch nanocomposites increased from 0.26 GPa and 0.32 GPa to 0.38 GPa and 0.44 GPa, respectively, for plant-CNC and tunicate-CNC when exposed to the magnetic field. The improved orientation and alignment of CNC in the presence of MF is further supported by Raman and scanning electron micrographs studies [23]. Linearly interconnected spherical fillers were also developed in polymer composites containing the Al_2_O_3_-Fe_3_O_4_ hybrid. Under the influence of a magnetic field, particles form thermal networks ensuring high thermal conductivity. Polymer composites obtained from Al_2_O_3_-Fe_3_O_4_ hybrid particles had high thermal conductivity in a direction parallel to the magnetic field force line with low filler content. It increased by more than 240% compared to composites with randomly dispersed fillers formed without the participation of a constant magnetic field [24,25,26,27,28,29,30].

The effect of a constant magnetic field on the gradient structure of aluminum warp composites (Al-21% b.w. Si and Al-40% b.w. Cu, where b.w. means “by weight”) was also studied during their directional solidification. The results of the experiment show that the application of a constant magnetic field during directional coagulation causes the formation of a gradient structure. The field-forced flow significantly changes the structure of the directionally solidified aluminum composite matrix exposed to the magnetic field [31,32]. Most polymers, including commercial synthetic polymers and biopolymers such as proteins, DNA, and polysaccharides, are non-magnetic materials and are believed to be inert to magnetic fields. In reality, however, they are magnetized, although poorly (they are diamagnetic), and therefore can react to the applied magnetic fields. Such a magnetic reaction of polymers can be used in polymer processing. For example, polymer particles (e.g., polystyrene and polypropylene) can be separated using a difference in their magnetic susceptibility; polymer fibers of sizes ranging from nanometers to micrometers can be lined up owing to their magnetic anisotropy; small polymer particles can be arranged into a designed pattern in spatially modulated magnetic fields; and magnetic fields can promote the formation of block copolymer microstructures. The ferromagnetic properties of magnetite (Fe_3_O_4_) and therefore its susceptibility to the external constant magnetic field led us to use this compound as an admixture to the polymer matrix. The composites prepared according to the procedure, still in the liquid state, were placed between the poles of an electromagnet (0.5 T). During the polymerization process, ferromagnetic magnetite particles were arranged along the magnetic field force lines. Other particles, even those diamagnetic, found in the spaces between ferromagnetic magnetite were also partially ordered, even though they are not as susceptible to the external magnetic field because they do not contain permanent magnetic dipoles in their structure. The polymerization of composites conducted in a constant magnetic field environment allowed us to expect that the materials obtained in this way would have new and different properties. The assumptions of our research based on a literature review were confirmed by the results of the conducted experiments. A special edition of the *Polymers* journal was devoted to this issue. That special edition was intended to document the latest advances in the use of magnetic fields for polymer processing and the production of functional polymer systems [33,34,35,36,37]. A group of German scientists have invented a polymer with shape memory which can be triggered by a magnetic field. To date, it was possible to activate shape changes in shape-memory materials by heat or radiation. Unfortunately, the use of heat is difficult or impossible in many areas, especially in medicine. The findings were published in the american journal [38]. Owing to the use of magnetic nanoparticles, they were able to control the shape of these polymers. This paves the way for new applications in the field of medical technology. Shape-memory polymers have the ability to return to their original shape after momentary deformation. Iron oxide nanoparticles were used in the process. They were dispersed in the polymer and then they converted the energy of the magnetic field into heat. As soon as the magnetic field was activated, the sample temperature began to rise. They used polyurethane (Tecoflex) and a biodegradable block polymer (PDC) in their experiments. The appropriate temperature could be achieved by changing the density of nanoparticles and by changing the magnetic induction value. The conclusions of the quoted article may explain the nature of the magnetic field impact on materials, including polymer composites [39]. Magnetic fields can affect the properties of organic molecules, and the effects of their action vary. These include Zeeman nuclear splitting induction, polaron Zeeman splitting, organic spintronics, and organic magnetoresistance. A polaron is a quasiparticle formed as a result of a local deformation of the crystalline network caused by electrostatic interaction, resulting from movement of a charged particle in the crystal. A magnetic polaron is a counterpart to a magnetic field where we are dealing with polarization of a magnetic center by a particle. The effect of the magnetic field on the aromatic molecule is the effect on the current of the aromatic ring, which can be regarded as an induction of a circular electron current π when a magnetic field perpendicular to the π is applied. The authors found that the photophysical properties of model phthalocyanine compounds and their aggregates show clear dependence on the magnetic field. This article also clarifies changes that occur under the influence of the magnetic field [40]. The authors constructed density functional formalism adapted to homogeneous external magnetic fields, which is the intermediary between conventional density functional theory and current density functional theory. The density functional is a series of quantum mechanical methods used to model the structure of chemical molecules or crystals. In the intermediate theory, referred to as linear vector potential, the basic variables are density, canonical momentum moment, and paramagnetic contribution to magnetic moment.

Ashkan et al. [41] synthesized bentonite/Fe_3_O_4_ nanocomposites by combining magnetic nanoparticles and bentonite in the presence and in the absence of an external magnetic field. The synthesis of nanocomposites was characterized, among others, by scanning electron microscopy (SEM) and X-ray diffraction (XRD). The application of a magnetic field during the synthesis process resulted in an increase in approximately 100% in the value of the specific loss power (SLP) of the nanocomposites compared to the sample synthesized without the magnetic field. This method can be used to achieve a higher SLP value, which is beneficial to the hyperthermia applied in the treatment of cancer.

Using molecular dynamics computer simulations, Zverev et al. [42] investigated how the application of a magnetic field affects the shape, integrity, and internal structure of clusters created by Stockmayer’s supercolloid magnetic polymers (SMPs). They observed deformation and the strongest monomer rearrangements from a liquid to a local hexatic order of the clusters formed by ring-like SMPs. However, clusters consisting of Y- and X-type ring-like SMPs demonstrate the highest magnetic susceptibility. Clusters formed by ring-like SMPs are generally not affected by the magnetic field. Majewski et al. [43] used a constant magnetic field to control the arrangement of Li-doped lamellar polyethylene oxide (PEO) microdomains in a liquid crystalline diblock copolymer over large length scales (>3 mm), building up the electrolytic membrane. The ordering of microdomains increases the membrane conductivity to about 50%. The effect of a constant magnetic field on elastomers was observed by Umehara et al. [36]. They produced a mono-link using bimodal magnetic elastomers, which showed marked changes in the elastic modulus under the influence of magnetic fields. The elastic modulus for bimodal magnetic elastomers can vary from 2.2 × 10^5^ to 1.7 × 10^6^ Pa in a magnetic field with an induction of 500 mT and without the presence of a magnetic field. Compression tests of up to 20% strain also demonstrated that the on-field tress for the bimodal magnetic elastomer was 1.24 times higher than the stress outside the magnetic field. Neodymium magnets, generating magnetic fields of approximately 1 T, are readily available for laboratory use and are widely used in everyday applications such as mobile phones and electric vehicles. Such widespread access to magnetic fields—unexpected 30 years ago—has helped scientists to discover new magnetic phenomena and use them for processing of diamagnetic materials. Although diamagnetism is well known, it is only in the last 30 years that scientists have applied magnetic treatment to different classes of diamagnetic materials such as ceramics, biomaterials, and polymers. The magnetic effects described by Yamato and Kimura [44] can be largely attributed to magnetic force, magnetic torque, and magnetic enthalpy, which, in turn, come directly from the well-defined magnetic energy. The orientation of crystalline polymers under the influence of an external, constant magnetic field is an example of a more complex magnetic effect.

## 2. Materials and Methods

### 2.1. Preparation of Components

The components for preparation of polymer composites included the following chemical reagents: Polylactide (PLA), EasyFil^TM^ PLA, Formfuture, Amsterdam, Netherlands; Epidian 5 (epoxy resin), Organika-Sarzyna, (Nowa Sarzyna, Poland); Hardener IDA, Organika-Sarzyna, (Nowa Sarzyna, Poland); Magnetite (Fe_3_O_4_)[Iron (II, III)oxide] 97%, Alfa Aesar, CAS: 1317-61-9; Avicel PH-1010 (crystalline cellulose), Sigma-Aldrich, Merc Life Science Sp. z o.o., Poznań, Poland; Colloidal Starch, 50 µm, Sigma-Aldrich, Merc Life Science Sp. z o.o., Poznań, Poland. The composites based on Epidian 5 and PLA polymers contained 10%, 20% and 30% b.w. magnetite (Fe_3_O_4_) and crystalline cellulose (Avicel PH-101, (Sigma-Aldrich, Merc Life Science Sp. z o.o., Poznań, Poland) admixtures also in the amount of 10%, 20%, and 30% b.w. Samples with a mass starch content of 10% have only been examined additionally, in comparison with analogous samples with a 10% mass content of crystalline cellulose.

### 2.2. Preparation of Test Samples and Testing Procedure

They were prepared according to the following procedure: a. develop the composition of the samples and the procedures for their preparation, b. weigh the individual components of polymer composites, c. add the individual components in the correct order (e.g., Epidian 5, magnetite and finally the catalyst) and their mechanical mixing at a speed of 300 revolutions per minute for a period of 180 s, d. place liquid polymer composites in previously prepared molds according to the PN-EN ISO 10210:2018-1 standard, e. place some of the samples between electromagnet poles at set magnetic induction of B = 0.5 T, for the polymerization period, f. leave the other part of the samples with an analogous composition for polymerization outside the electromagnet (without exposure to a magnetic field). The measurement of each sample was repeated five times. The results in the tables are the mean values of these five measurements. Magnetite (Fe_3_O_4_) orientation during the polymerization reaction was already in line with the direction of the magnetic induction vector B, as orientation occurred at the beginning (first minute) when the magnetic field was switched on, while polymerization occurred after 90–120 min. During this time, the magnetic field worked. Polymerization occurred only after 90–120 min, therefore, there was a sufficient time for the orientation of magnetite to be as consistent as possible with the direction of the magnetic induction vector, regardless of the difference in the viscosity of the solutions. Magnetite orientation was influenced by the magnetic field intensity value, but the article showed changes in the magnetic field based on one optimal magnetic field intensity value selected on the basis of previous studies. We wanted to focus on changes in individual properties in the magnetic field rather than on quantitative changes dependent on the value of the magnetic field intensity. Measurement errors of the individual parameters were within the ranges: for water absorption capacity ±0.003%, for frost resistance ±0.002%, for chemical resistance ±0.005%, for mechanical strength ±0.1 MPa, and for impact strength ±0.01 kJ/m^2^.

### 2.3. Testing Methodology

The research was conducted using the FEI Quanta 3D Field Emission Gun Scanning Electron Microscopy (FEG-SEM, FEI Company, Hillsboro, OR, USA) equipped with an X-ray spectrometer with EDAX Genesis energy dispersion (Mahwah, NJ, USA). The parameters used for measurements were as follows: the electron beam acceleration voltage: 10 kV (for PLA), 6 kV (for Ep 5), the electron beam current: 93.3 pA (for PLA), 32 nA (for Ep 5), the distance between the electron beam focus point and the objective lens pole piece (working distance): 9.6–10.3 mm (for PLA), 9–10 mm (for Ep 5), magnification: 250–2000× (for PLA), 250–8000× (for Ep 5), detectors: ETH (Everhart–Thornley secondary electron detector) and vCD (backscattered electron detector for low acceleration voltages) for PLA as well as ETH and BSE (backscattered electron detector) for Ep 5. Measurements were also made using a Bruker D8 Discover X-ray diffractometer (Bruker AXS Inc., Madison, WI, USA) with a Euler’s disk and an x-y-z table. The parameters used for the measurements were as follows the beam radiation and optics were filtered Co Kα-series polycalypillary primary beam optics with a pinhole collimator of φ 1 mm; for the study of the crystallographic texture, parafocusing secondary beam optics were used with position-sensitive LynxEye semiconductor detector (Bruker AXS GmbH, Karlsruhe, Germany) with a span of 2.6° in the 2ϴ angle space; and for the study of the stress state, parallel secondary beam optics were used with a Soller collimator with the equatorial divergence of 0.23°. Mechanical tensile and bending strength tests were conducted on a Zwick/Roell Z050 mechanical strength test machine (Zwick Roell, Ulm, Germany), KL 0.05 with a 50 kN measuring head. A QC 639F type Charpy hammer (Cometech Testing Machines Co., Ltd., Taichung City, Taiwan) with a pendulum weight of 5 J and a 2.9 m/s pendulum impact on the sample speed was used for the impact strength test of the samples. The constant magnetic field within the B = 0–1.2 T magnetic induction range was produced by means of a laboratory electromagnet ER—2505 with a teslameter and a PZP—80 type control device.

## 3. Results and Discussion

Based on the literature and previous self-conducted studies describing the effects of a constant magnetic field on various test objects, changes in mechanical properties (increased strength) as well as physical and chemical properties (increased frost resistance, decreased water absorption, increased chemical resistance) of polymer composites obtained in the magnetic field environment were also expected. In addition, a special edition of the journal “*Polymers*” (Special Issue “Magnetic Field in Polymer Research”, Prof. Tsunehisa Kimura, Prof. Masafumi Yamato) of 2018–2019, which presented a number of papers, indicated such positive changes.

### 3.1. Effect of the Type of Polymer Used on Change in the Properties of the Resultant Composites in a Constant Magnetic Field and without a Magnetic Field

**Figure 1:**
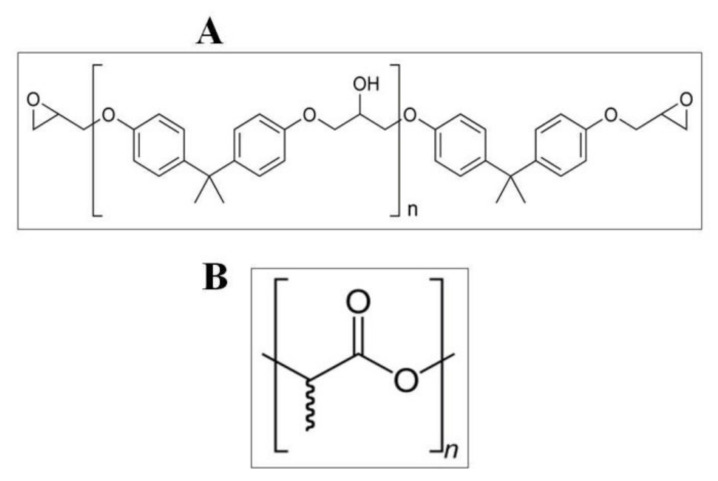
Structure of epoxy resin (**A**) and polylactide (**B**).

At the beginning of the study, it was decided that there should be an observation on how the type of polymer used affects the change in the properties of the resulting composites in a constant magnetic field and without a magnetic field. Two polymers were used: polylactide (PLA), a fully biodegradable polymer belonging to the aliphatic polyester group, and epoxy resin (Epidian 5), whose components are polyphenols (polyglycols) and epichlorohydrin, or oligomers containing epoxy groups (Figure 1).

**Figure 2:**
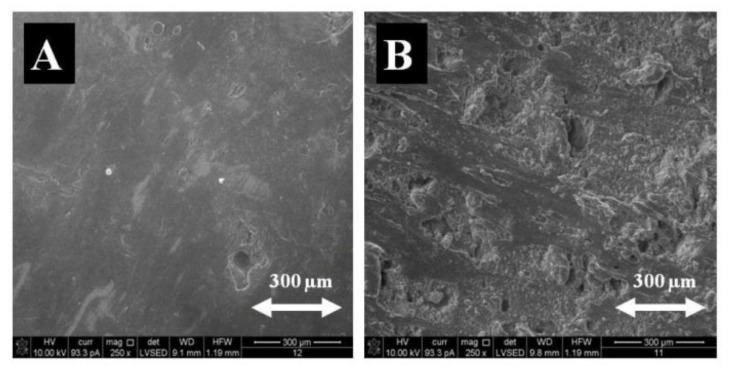
Microstructure of PLA and Fe_3_O_4_ composite polymerized without CMF (**A**) and under the exposure to CMF with magnetic induction B = 0.5 T (**B**), image of secondary electrons SE, magnification 250×.

The admixtures to the composites, added in the amount of 10%, 20%, and 30% by weight, were magnetite (Fe_3_O_4_), and crystalline cellulose (Avicel PH-1010), as well as starch (a polysaccharide), added in the amount of 10% by weight. Surface morphology studies were conducted by SEM (microstructure studies), using the FEI Quanta 3D FEG-SEM scanning electron microscope, with EDAX Genesis energy dispersion at the magnification of 250, 650, 1000, and 2000 times. The microstructure of samples polymerized in a constant magnetic field (CMF) and without a magnetic field was recorded by means of secondary electrons (Figure 2).

As indicated by the surface analysis at (B = 0 T), there were a lot of admixture clusters, small in size, fairly and regularly distributed on the composite surface. At (B = 0.5 T), the admixture aggregates of irregular shapes differing in size (from very small to very large ones) were observed. When analyzing the surface composition of PLA-based composites, it can be concluded, based on the two test areas, that the composites generated in CMF (B = 0.5 T) contain less carbon (C), less oxygen (O), and more iron (Fe) than the composites obtained without a magnetic field (B = 0 T) (Table 1).

**Figure 3:**
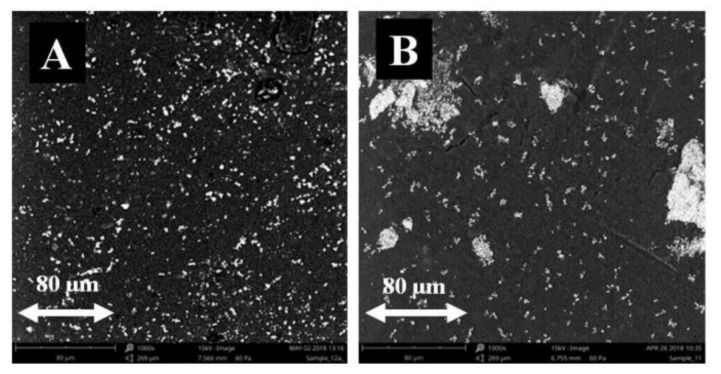
Microstructure of PLA and Fe_3_O_4_ composite polymerized without CMF (**A**) and under the exposure to CMF with magnetic induction B = 0.5 T (**B**), magnification 1000×.

The measurements were repeated on a Phenom XL brand scanning electron microscope (SEM) with an integrated EDS detector (Thermo Fisher Scientific, Waltham, MA, USA), at a magnification of 1000 times (Figure 3). This allowed for the drawing of similar conclusions as in the previous studies.

**Figure 4:**
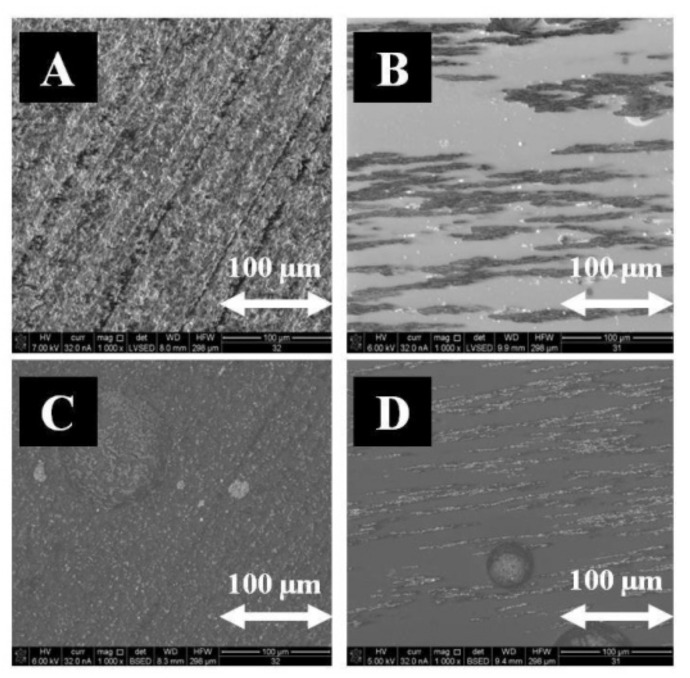
Microstructure of epoxy resin (Ep 5) and Fe_3_O_4_ composite polymerized without the application of CMF (**A**,**C**) and in CMF of B = 0.5 T (**B**,**D**), image of secondary electrons SE (**A**,**B**) and image of backscattered electrons BSE (**C**,**D**), magnification 1000×.

Comparative studies were conducted using a polymer in the form of epoxy resin (Epidian 5). Magnetite (Fe_3_O_4_) was also used as an admixture. The microstructure of samples polymerized in CMF and without a magnetic field was recorded by means of secondary electrons and backscattered electrons using the FEI Quanta 3D FEG-SEM scanning electron microscope (FEI Company, Hillsboro, OR, USA), with EDAX Genesis energy dispersion (Mahwah, NJ, USA, Figure 4).

**Figure 5:**
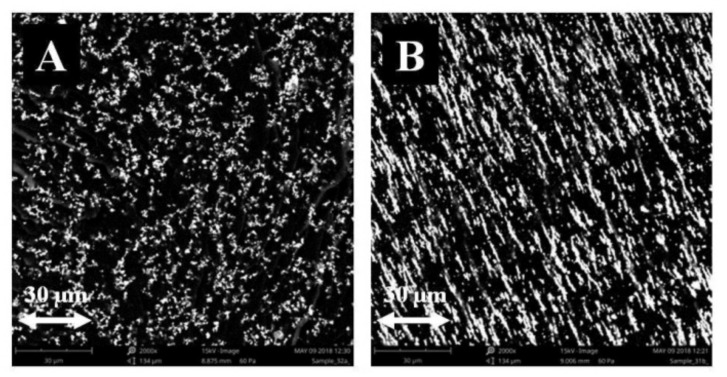
Microstructure of epoxy resin composite (Ep 5) and Fe_3_O_4_ polymerized without CMF (**A**) and in CMF of magnetic induction B = 0.5 T (**B**), magnification 2000×.

The measurements were repeated on a Phenom XL brand scanning electron microscope (SEM) with an integrated EDS detector, at a magnification of 2000 times (Figure 5).

When analyzing the surface composition of composites based on (Epidian 5), it can be concluded that the composites formed in CMF (B = 0.5 T) also have less carbon (C), less oxygen (O), and more iron (Fe) than the composites produced without a magnetic field (B = 0) (Table 2).

**Figure 6:**
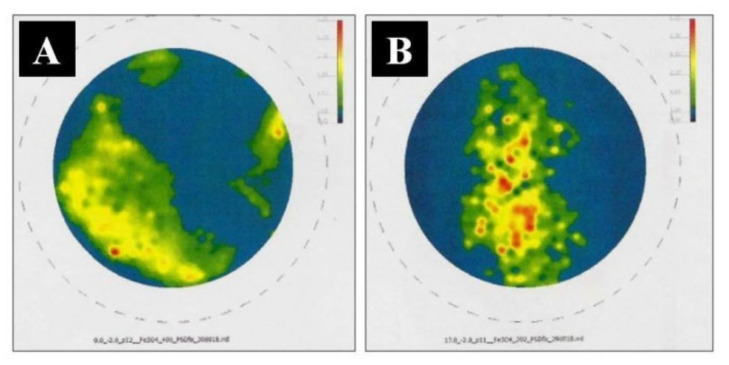
Corrected, incomplete polar figure obtained for the Fe_3_O_4_ phase, for [Polylactide (PLA) and magnetite (Fe_3_O_4_)] composite without the application of CMF (**A**) and in CMF of B = 0.5 T (**B**).

The tests were also conducted using XRD X-ray diffraction (stress state testing and crystallographic texture determination) using a Bruker D8 Discover X-ray diffractometer (Bruker, Billerica, MA, USA). For the [(PLA) and (Fe_3_O_4_)] composites obtained without the application of CMF and in CMF of B = 0.5 T, incomplete polar figures were obtained for the Fe_3_O_4_ phase. A heterogeneity of the intensity of the recorded diffraction signals is visible (Figure 6). No privileged crystallographic orientation of either the polymer (PLA) phases or the Fe_3_O_4_ phases were observed. The own stress state was determined for the Fe_3_O_4_ phase based on the observations of reflection of 440 Fe_3_O_4_ (E = 178 GPa, v = 0.33). It can therefore be assumed that the phases considered demonstrate stresses of σ = 15–25 MPa (for B = 0 T) and σ = 0–20 MPa (for B = 0.5 T), and are of a powder-like nature.

**Figure 7:**
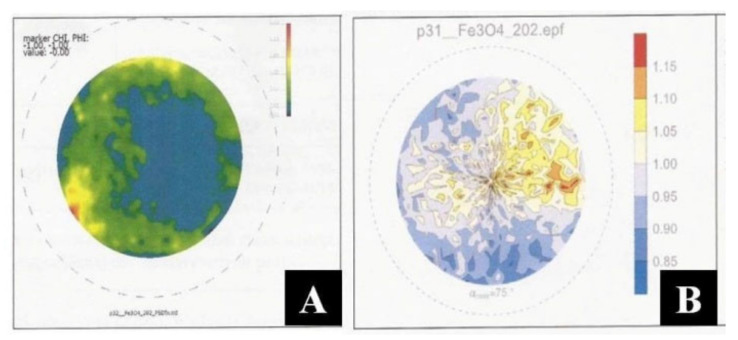
Corrected, incomplete polar figure obtained for the Fe_3_O_4_ phase, for [epoxy resin (Epidian 5) and magnetite (Fe_3_O_4_)] composite without the application of CMF (**A**) and in CMF of B = 0.5 T (**B**).

For the [(Epidian 5) and (Fe_3_O_4_)] composites produced without the application of CMF and in CMF of B = 0.5 T, incomplete polar figures were obtained for phases Fe_3_O_4_. A heterogeneity of the intensity of the recorded diffraction signals is visible (Figure 7). No privileged crystallographic orientation of either the polymer (Epidian 5) phases or the Fe_3_O_4_ phases was observed. The own stress state was determined for the Fe_3_O_4_ phase based on the observations of reflection of 440 Fe_3_O_4_ (E = 178 GPa, v = 0.33). The phases concerned show σ = 10–15 MPa (for B = 0) and σ = −18–25 MPa (for B = 0.5 T), and are powder-like in character.

The obtained composites were subjected to physico-chemical tests such as water absorption and frost resistance tests. Absorptivity, i.e., the ability of a composite to absorb water, was calculated using the following Equation (1):

(1) $$n_{w}=\frac{\left(m_{n}-\;m\right)}{m}\times 100\%$$

where: m_n_ is the mass of the sample saturated with distilled water [g] and m is the mass of dry sample [g].

Studies of selected composites are presented in Table 3, which demonstrates that CMF with magnetic induction B = 0.5 T increases the absorbency of the composites, both based on (PLA) and (Epidian 5).

Statistical analyses demonstrated that the measurements were made with a 3% error.

There are many different methods for testing frost resistance. The methods for determining the resistance to freezing and defrosting are contained in international standards: PN-EN 772-18:2011; MON-EN 771-2:2011; MON-EN 1338:2005. The frost resistance tests presented in the article were based on PN-EN 206 + Al:2016 and the national supplement PN-88/B-06250, which describes the so-called normal frost resistance test, based on the test of mass loss due to repeated freezing and defrosting of samples. In our article, the frost resistance was defined as a percentage of sample loss weight due to the damage caused by freezing water in this sample, such as cracks and chips (Equation (2)). Thus, the lower damage of the sample, the lower weight loss, and, consequently, the higher frost resistance. In the material science, the sample weight loss and the mechanical properties were often used to measure the frost resistance.

Frost resistance, i.e., the determination of mass loss under the influence of temperature T = −20 °C was calculated from the following Equation (2):

(2) $$S=\frac{\left(m_{1}-{\;m}_{2}\right)}{m_{1}}\times 100\%$$

where m_1_ is the mass of the dried sample prepared for testing [g] and m_2_ is mass of the dried sample at the end of the test [g].

Studies of selected composites are presented in Table 4, which shows that CMF with a magnetic induction B = 0.5 T reduces the weight loss in the composites and increases their frost resistance, with respect to the composites based on both PLA and Epidian 5 polymers.

Statistical research showed that the measurements were performed with an error of approx. 2%. In the course of our measurements, we also calculated the error made in the tests (confidence interval). The identical measuring sample was repeated five times. Let us follow the statistical analyses using the measurement example from Table 4 (Frost Resistance of Composites) for Epidian 5, with magnetic induction B = 0 T. The mean value was 0.07959%. The measurement error, e.g., for frost resistance tests, was ±0.002%. The obtained consecutive frost resistance measurements were as follows: 0.07791; 0.08022; 0.08104; 0.07859; and 0.08021%. From these measurement values we calculated the arithmetic mean $\bar{x}$, as the value approximating most closely the actual value (such as presented in the measurement tables):

(3) $$\bar{x}=(x_{1}+x_{2}+x_{3}+\ldots \ldots +n)/n=1/n\;\sum_{i=1}^{n}x_{i}\;\mathrm{for}\;n=5\;\bar{x}=0.07959$$

Then, we calculated variance σ^2^, i.e., the value characterizing the dispersion, the deviation of the individual results from the actual value:

(4) $$\sigma^{2}=1/(n-1)\;\sum_{i=1}^{n}(x_{i}-\bar{x}{)}^{2}\;\mathrm{for}\;n=5\;\sigma^{2}=1/4\;\sum_{i=1}^{5}(x_{i}-\bar{x}{)}^{2}\;\sigma^{2}=1.67655\;\times \;10^{-6}$$

To determine the measure of an error affecting a single measurement, we calculated the standard deviation (average square error) σ:

(5) $$\sigma =\{\;\sum_{i=1}^{n}\{(x_{i}-\bar{x}{)}^{2}/(n-1)]{\}}^{1/2}\;\mathrm{for}\;n=5\;\sigma =1/2\;[\sum_{i=1}^{5}{\left(x_{i}-\bar{x}{)}^{2}\right]}^{1/2}\;\sigma =1.29482\;\times \;10^{-3}$$

Using the previous parameters, we can calculate the variation coefficient ν, i.e., the normalized measure of dispersion:

(6) $$\nu =\sigma /\bar{x}\;\times \;\nu =1.627\times \;10^{-2}=0.01627$$

On the other hand, to determine the measure of error carried by the arithmetic mean in relation to the unknown actual measured value, we calculated the mean average error (mean squared error of the arithmetic mean) S$\bar{x}$:

(7) $$S\bar{x}=\sigma /(n{)}^{1/2}\;\mathrm{for}\;n=5\;S\bar{x}=5.79061\;\times \;10^{-4}$$

The arithmetic mean of the measurements $\bar{x}$ differs from the unknown actual (measured) value μM. This difference is described by the t-value:

(8) $$t=|\bar{x}-\mu M|/S\bar{x}$$

is subject to the Student’s distribution, which depends on the number of measurements and the values obtained as a result of the tests:

S (t, k) = c (k) [1 + (t^2^/k)]^−(k + 1)/2^ (9)

where n = number of measurements, k = number of degrees of freedom (k = n − 1).

The difference between the actual value of μM, and the mean $\bar{x}$ (the range of random measured values around the obtained mean value within which the actual value falls) is characterized by the width of the confidence interval ε:

(10) $$\varepsilon =(\bar{x}-\mu M)=t\;\times \;S\bar{x}\;\varepsilon =2.76\;\times \;5.79061\;\times \;10^{-4}=1.60747\;\times \;10^{-3}$$

The value of function t can be read from statistical tables, for the specified k-value, and for the significance level value. The confidence level P amounts to:

P = 1 − α(11)

In our studies, the number of measurements is n = 5, so the number of degrees of freedom k = 4. We assumed 0.05 as the significance level; thus, the confidence level P was 0.95, which means that the actual values fall within the given range with a probability of 95%. The t-value read from the Student’s distribution tables was 2.776. The results could therefore be presented ultimately as the arithmetic mean of the measurements and the width of the confidence interval:

(12) $$(\bar{x}\;\pm \;\varepsilon )\;\bar{x}=0.07959\;\pm \;0.00161$$

A similar reasoning can be applied to all the measurements contained in the tables when calculating the width of the confidence interval for each measurement.

### 3.2. Effect of the Type of Filler Added to the Polymer on Change in the Properties of the Resultant Composites Obtained in a Constant Magnetic Field and without a Magnetic Field

**Figure 8:**
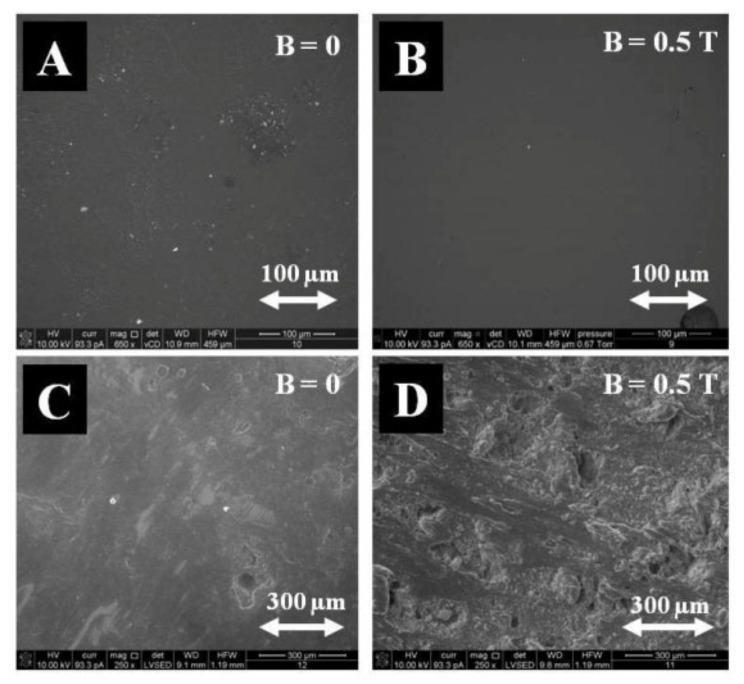
Microstructure of PLA and starch composite polymerized without CMF (**A**) and with the application of CMF (**B**), the image of backscattered electrons BSE as well as PLA and Fe_3_O_4_ composite polymerized without the application of CMF (**C**) and with the application of CMF (**D**), magnification 650× and 250×.

Since magnetite filler in epoxy resin matrix is known as an effective microwave radiation absorber [45,46], the (Epidian 5—magnetite) composite can be used in the “stealth” technology which enables the hiding of various objects both from sight and various devices using radar waves or thermovision and shields the material for electromagnetic radiation. To the best of our knowledge, the (Epidian 5—magnetite) composite is the first example of an epoxy resin composite with magnetite powder as a filler that has been cured in a constant magnetic field. Its mechanical properties, such as Young’s module, bending stress, and impact strength were improved compared to those of the samples prepared without a constant magnetic field. The studies compared the microstructure of PLA polymer-based with starch admixture and Fe_3_O_4_-based composite samples polymerized in CMF and without a magnetic field recorded using backscattered electrons with the FEI Quanta 3D FEG-SEM scanning electron microscope, with EDAX Genesis energy dispersion (Figure 8).

When analyzing the surface composition of PLA polymer-based with starch admixture and Fe_3_O_4_-based composites, it can be concluded that composites produced in CMF (B = 0.5 T) have lower carbon content (C) in both types of composites, less oxygen (O) in the case of the (PLA and Fe_3_O_4_) composite, and more in the case of the (PLA and starch) composite compared with the samples obtained without the influence of a magnetic field (Table 5).

The resulting composites were also subjected to physico-chemical tests such as water absorption and frost resistance tests. Studies of selected composites are presented in Table 6, which shows that CMF with a magnetic induction B = 0.5 T increases the absorbency of composites, most significantly in the case of those with the addition of Fe_3_O_4_, less of those with the addition of starch and the least of those with the addition of cellulose (Avicel PH-1010).

Statistical analyses demonstrated that the measurements were made with a 3% error.

Frost resistance studies of the selected composites are presented in Table 7, which demonstrates that CMF with a magnetic induction B = 0.5 T reduces the mass loss in composites and increases their frost resistance; most for those with an admixture of starch, less for those with an admixture of Fe_3_O_4,_ and even less for those with cellulose (Avicel PH-1010).

Statistical analyses demonstrated that the measurements were made with a 2% error.

Mechanical strength studies were also performed, including bending, tensile strength, impact strength tests and Young’s modulus of epoxy resin-based polymer composites (Epidian 5) with an admixture of crystalline cellulose (Avicel PH-1010) and magnetite (Fe_3_O_4_). Mechanical tensile strength is the highest stress that a material sample can withstand when stretched. This stress is determined by means of the following Equation (13):

(13) $$R_{r\;}=\frac{F_{r}}{A}$$

where F_r_ is the maximum tensile force(N) and A is the section of the stretched sample, perpendicular to the direction of the force action (cm^2^).

The tests are presented in Table 8, where: σ_m_ is the stress in the material during stretching and ε_m_ is the elongation at stretching (deformation). The tests were conducted according to the DIN EN ISO 527-1 standard, the initial force was 0.1 N, the test speed was 50 mm/min, and the distance of the handles with the sample mounted to 60 mm.

The mechanical tensile strength of pure epoxy resin Ep5 was 20.1 MPa for B = 0 T and 18.6 MPa for B = 0.5 T. The addition of Avicel (powdered cellulose) in the amount of 10% to Ep5 practically did not change the tensile strength of the composite (23.8 MPa for B = 0 T and 20.7 MPa for B = 0.5 T). Magnetite (Fe_3_O_4_) weakens the tensile strength compared to Avicel. The tensile strength is 16.2 MPa for B = 0 T and 12.9 MPa for B = 0.5 T. Young’s modulus, i.e., the elasticity modulus E (Table 9), was also determined. It is the ratio of the normal stress σ_m_ to elongation (or shortening) ε_m_. It is a measure of the material rigidity, i.e., the inclination angle of the σ-ε line. It is determined at one deformation speed v (in our study, it was 50 mm/min).

For pure Ep5 resin, Young’s elasticity modulus amounted to 1.32 N/m^2^ for B = 0 T and 1.16 N/m^2^ for B = 0.5 T). Ep5 with Avicel (10%) had a modulus of 1.49 N/m^2^ at (B = 0 T) and 1.22 N/m^2^ at (B = 0.5 T). Magnetite admixture (10%) caused the Young’s modulus to increase in the magnetic field, from 1.31 N/m^2^ (B = 0 T) to 1.72 N/m^2^ (B = 0.5 T). The mechanical bending strength of the selected composites was also examined (Table 10). This is the highest stress that a sample of material can withstand when bent. This stress is determined by the following Equation (14):

(14) $$R_{z\;}=\frac{M}{W}$$

where M is the bending moment (Nm or kGcm) and W is the bent element cross-sectional strength indicator (cm^3^),

(15) $$M=\frac{F\;\times l}{4}$$

where F is the destructive force (kG) and l is the beam span between the supports (cm),

(16) $$W=\frac{b\;\times {h\;}^{2}}{6}$$

where b is the beam width (cm) and h is the beam height (cm).

The samples were tested according to DIN EN ISO 178, with the initial force of 0.1 N, test speed of 5 mm/min, and a bending module speed of 1 mm/min. The sample parameters were: height h = 6 mm, width b = 16 mm, and length l = 60 mm.

The weakest mechanical bending strength was demonstrated by pure Ep5 resin (stress value of 12.6 MPa, elongation of 3.3%). A 10% Avicel (powdered cellulose) admixture increased that bending strength from 12.6 to 50.7 MPa. A 10% magnetite (Fe_3_O_4_) admixture had a less beneficial effect on the bending strength value of 30.6 MPa. The composites were also studied using the Charpy impact test (Table 11). The tests were conducted with a QC 639F type Charpy hammer, a pendulum mass of 5 J, a pendulum impact speed of 2.9 m/s. The formula: U = L/A (J/m^2^), where U is the impact strength, L is the work needed to break the standardized sample (J), and A is the area of the sample cross-section at the site of the notch (m^2^).

There was an increase in the impact strength of pure Ep5 resin in CMF (from 2.61 to 2.94 kJ/m^2^). A 10% addition of a diamagnetic (cellulose) caused a slight increase in the effect of CMF on the impact strength of the composite (from 3.78 to 3.82 kJ/m^2^) but increases the impact strength compared to Ep5. A 10% admixture of magnetite resulted in a greater increase in the impact strength of the composite in CMF (from 3.68 to 4.94 kJ/m^2^). Through additional components, in this case, the introduction of biological material into the polymer matrix, a controlled change in selected mechanical, physical, and chemical properties can be achieved. However, according to the principle, the more material introduced, relative to the quantity and type of polymer, to a particular manufacturing technology, the greater the degree of degradation that can be obtained. This is described by characteristic values, in accordance with the accepted test methods, e.g., strength, elongation, brittle cracking, the occurrence of specific function groups or their absence determined by instrumental methods, e.g., ATR-FTIR, etc. The biodegradability property for polymer-filler composites was assumed on the basis of the available literature concerning the above topic. The research described in the paper did not carry out additional biodegradation studies, and adopted a general thesis based on studies such as [47,48]. In the paper, we may have concluded too quickly that typical biological degradation would be obtained, and we were generally concerned with degradation, that is, the design and development of a product that, due to the need to protect the environment, is not made in 100% of plastic, but is filled to a large extent with a biological filler. The difference in biodegradability depending on the magnetic field intensity is significantly smaller than the impact of the amount and type of composite components.

### 3.3. Effect of the Percentage of Filler Added to the Polymer on Change in the Properties of the Resultant Composites in a Constant Magnetic Field and without a Magnetic Field

The mechanical tensile strength was presented for epoxy resin-based composites (Epidian 5) with the admixture of magnetite (Fe_3_O_4_) or crystalline cellulose (Avicel PH-1010) in the quantities of 10%, 20% or 30% by weight. The tests are shown in Table 12, where σ_m_ is the stress in the material being stretched. The tests were conducted according to DIN EN ISO 527-1, the initial force was 0.1 N, the test speed was 50 mm/min, and the distance between the handles with the sample mounted 60 mm.

The tensile strength of pure Ep5 epoxy resin is 20.1 MPa for B = 0 T and 18.6 MPa for B = 0.5 T. The addition of Avicel (powdered cellulose) in the amount of 10% to Ep5 practically did not affect the tensile strength of the composite (23.8 MPa for B = 0 T and 20.7 MPa for B = 0.5 T). Magnetite (Fe_3_O_4_) weakened the tensile strength compared to Avicel. The tensile strength was 16.2 MPa for B = 0 T and 12.9 MPa for B = 0.5 T. Young’s modulus, i.e., the elasticity modulus E, was also determined (Table 13). It is the quotient of the normal stress σ_m_ to elongation (or shortening) **ε_m_**. It is a measure of the rigidity of the material, i.e., the σ-ε line inclination angle. It is determined at one deformation speed (in our study it was 50 mm/min).

For pure Ep5 resin, the value of Young’s elasticity modulus was 1.32 N/m^2^ for B = 0 T and 1.16 N/m^2^ for B = 0.5 T. Ep5 with Avicel (10%) had a modulus of 1.49 N/m^2^ at (B = 0 T) and 1.22 N/m^2^ at (B = 0.5 T). The addition of magnetite (10%) caused the Young’s modulus to increase from 1.31 N/m^2^ (B = 0 T) to 1.72 N/m^2^ (B = 0.5 T). The mechanical bending strength of epoxy resin (Epidian 5) composites with magnetite (Fe_3_O_4_) or crystalline cellulose (Avicel PH-1010) admixture in the amount of 10%, 20%, or 30% (Table 14) was also investigated. This is the highest stress σ_fM_ that a material sample can withstand when being bent. The samples were tested according to DIN EN ISO 178, with an initial force of 0.1 N, a test speed of 5 mm/min, and a bending module speed of 1 mm/min. The sample parameters were: height h = 6 mm, width b = 16 mm, and length l = 60 mm.

The lowest mechanical bending strength was demonstrated by pure Ep5 resin with a stress value of 12.6 MPa. A 10% addition of Avicel (powdered cellulose) increased the bending strength from 12.6 to 50.7 MPa. A less beneficial effect on the value of a bending strength of 30.6 MPa was observed for the composite with a 10% magnetite (Fe_3_O_4_) admixture. The composites were also tested for impact strength by the Charpy method (Table 15). The tests were conducted using a QC 639F type Charpy hammer, a pendulum mass of 5 J, and a pendulum impact speed of 2.9 m/s.

There was an increase in impact strength of pure Ep5 resin in CMF (from 2.61 to 2.94 kJ/m^2^). A 10% addition of a diamagnetic (cellulose) caused a slight increase in the effect of CMF on the impact strength (from 3.78 to 3.82 kJ/m^2^) but increased the resistance impacts compared to Ep5. A 10% admixture of magnetite increased the impact strength of the composite in the CMF (from 3.68 to 4.94 kJ/m^2^). The composite samples were also tested for physico-chemical properties. Absorbency, i.e., the ability of the particular composite to absorb water, was investigated (Table 16).

Statistical analyses demonstrated that the measurements were made with a 3% error.

The frost resistance of these composites was also studied. A measure of frost resistance was the observed loss of mass of the composites (Table 17).

Statistical analyses demonstrated that the measurements were made with a 2% error.

The chemical resistance of the composites to an acid (H_2_SO_4_) and a base (NaOH) was also studied. The measure of chemical resistance to the acid (H_2_SO_4_) was the weight loss of the individual composites tested (Table 18).

The measure of chemical resistance to the base (NaOH) was also the weight loss of the tested composites (Table 19).

## 4. Conclusions

The article presents the studies of the polylactide polymer (PLA) and epoxy resin (Epidian 5) composites. They were polymerized in a constant magnetic field environment and without the influence of a magnetic field. The tested composites contained admixtures in the form of magnetite (Fe_3_O_4_) and crystalline cellulose (Avicel PH-1010) in the amount of 10%, 20% and 30% by weight, and starch in the amount of 10%. Changes in physico-chemical properties due to the action of a constant magnetic field with a magnetic induction value B = 0.5 T were observed. The changes were observed in tensile strength, bending strength, impact strength, water absorption tests, frost resistance, chemical resistance to acids and bases, and with SEM microscopic testing, EDS composite composition, and XRD structural studies. By analyzing the surface composition of polymer-based (PLA) as well as (Epidian 5) composites, it was found that composites produced in CMF (B = 0.5 T) contain less carbon (C), less oxygen (O) and more iron (Fe) than composites produced without the magnetic field (B = 0 T). On the basis of the XRD method, heterogeneity in the intensity of the recorded diffraction signals was found. No privileged crystallographic orientation of the polymer phase (PLA), (Epidian 5), or the Fe_3_O_4_ phases was observed. CMF with a magnetic induction B = 0.5 T was found to increase the absorbency of composites, both based on (PLA) and (Epidian 5).

This is not the case for frost resistance tests. A constant magnetic field with a magnetic induction B = 0.5 T reduces the mass loss, i.e., increases the frost resistance of the composites, both (PLA)- and (Epidian 5)-based. The addition of Avicel (powdered cellulose) in the amount of 10% to (Epidian 5) practically does not affect the mechanical tensile strength of the composite but increases the mechanical bending strength of the composite. It also causes a slight increase in the influence of CMF on the impact strength of such a composite. The addition of magnetite (Fe_3_O_4_) in the amount of 10% to the polymer (Epidian 5) causes the Young’s modulus for such composites to increase in a constant magnetic field environment.

As the value of magnetic induction B increases, the energy Em of the magnetic field acting on the polymer increases exponentially. The magnetic field, influencing the multiphase structure of the material, changes the state of thermodynamic equilibrium of the material as well. Thermodynamic functions such as enthalpy and internal energy can also be affected by the magnetic field. The internal energy of a diamagnetic material (polymer) decreases parabolically with an increase in the magnetic field, whereas the internal energy of ferromagnetics (iron oxide, iron carbonyl) does not change in the magnetic field. The enthalpy of a diamagnetic (polymer) grows parabolically as the magnetic field increases, while that of ferromagnetics (iron oxide, iron carbonyl) decreases linearly as the magnetic field grows. The change in internal energy U or enthalpy H under the magnetic field is due to the direction of charges in the material or ions in the liquid, as well as the disappearance of the insulating thermal barrier. To the best of our knowledge, the (Epidian 5—magnetite) composite is the first example of an epoxy resin composite with magnetite powder as a filler that has been polymerized in a constant magnetic field. The use of a constant magnetic field as an additional parameter contributes to solving the problem of the demand for materials with new modified and improved properties.

## Figures and Tables

**Table 1:** Composition (in % of bulk) of composite surfaces (PLA and Fe_3_O_4_) formed in constant magnetic field (CMF) (B = 0.5 T) and without a magnetic field (B = 0 T).

Magnetic Induction	Area Number	Carbon (C)	Oxygen (O)	Iron (Fe)
B = 0 T	1	48.58	49.30	2.12
	2	48.70	48.21	3.09
	$\bar{X}$	48.64	48.75	2.61
B = 0.5 T	1	43.66	44.20	12.26
	2	45.48	44.28	10.11
	$\bar{X}$	44.57	44.24	11.19

**Table 2:** Composition (in % b.w.) of the surface of (Ep 5) -based composites, formed in CMV (B = 0.5 T) and without a magnetic field (B = 0 T).

Magnetic Induction	Area Number	Carbon (C)	Oxygen (O)	Iron (Fe)
B = 0 T	1	66.90	26.98	6,12
	2	67.25	28.44	4.31
	$\bar{X}$	67.07	27.71	5.22
B = 0.5 T	1	63.81	24.94	11.25
	2	62.58	23.69	13.73
	$\bar{X}$	63.19	24.31	12.50

**Table 3:** Absorptivity of composites.

Ordinal Number	Type of Composite	Absorptivity (%)	
		B = 0 T	B = 0.5 T
1	Epidian 5	0.1224	0.1417
2	Epidian 5 + Fe_3_O_4_ (10% b.w.)	0.1014	0.2070
3	PLA	0.2780	0.4607
4	PLA + Fe_3_O_4_ (10% b.w.)	0.6232	1.4820

**Table 4:** Frost resistance of composites.

Ordinal Number	Type of Composite	Frost Resistance (%)	
		B = 0 T	B = 0.5 T
1	Epidian 5	0.07959	0.03450
2	Epidian 5 + Fe_3_O_4_ (10% b.w.)	0.09480	0.00022
3	PLA	0.21097	0.00101
4	PLA + Fe_3_O_4_ (10% b.w.)	0.08898	0.00024

**Table 5:** Composition (in % by weight) of [PLA and Fe_3_O_4_ (10% b.w.)] and [PLA and starch (10% b.w.)] composite surfaces formed in CMF (B = 0.5 T) and without a magnetic field (B = 0 T).

Magnetic Induction	Area Number	Carbon (C)	Oxygen (O)	Iron (Fe)	Sodium (Na)
B = 0 T	PLA and starch	58.27	41.03	-	0.70
	PLA and Fe_3_O_4_	48.64	48.75	2.61	-
B = 0.5 T	PLA and starch	47.88	51.93	-	0.19
	PLA and Fe_3_O_4_	44.57	44.24	11.19	-

**Table 6:** Absorptivity of composites.

Ordinal Number	Type of Composite	Average of Water Absorption (%)	
		B = 0 T	B = 0.5 T
1	Epidian 5	0.1224	0.1417
2	Epidian 5 + Avicel PH-1010 (10% b.w.)	0.2588	0.2791
3	Epidian 5 + Fe_3_O_4_ (10% b.w.)	0.1014	0.2070
4	PLA	0.2780	0.4607
5	PLA + starch (10% b.w.)	0.2246	0.8108
6	PLA + Fe_3_O_4_ (10% b.w.)	0.6232	1.4820

**Table 7:** Frost resistance of composites.

Ordinal Number	Type of Composite	Average of Frost Resistance (%)	
		B = 0 T	B = 0.5 T
1	Epidian 5	0.07959	0.03450
2	Epidian 5 + Avicel PH-1010 (10% b.w.)	0.22112	0.07115
3	Epidian 5 + Fe_3_O_4_ (10% b.w.)	0.09480	0.00022
4	PLA	0.21097	0.00101
5	PLA + starch (10% b.w.)	0.04176	0.01154
6	PLA + Fe_3_O_4_ (10% b.w.)	0.08898	0.00024

**Table 8:** Comparison of the mechanical tensile strength of composites.

Ordinal Number	Type of Composite	Magnetic Induction B (T)	Stress during Stretching σ_m_ (MPa)	Elongation When Stretching ε_m_ (%)
1	Epidian 5	0	20.1	2.0
		0.5	18.6	1.6
2	Epidian 5 + Fe_3_O_4_ (10% b.w.)	0	16.2	1.1
		0.5	12.9	0.75
3	Epidian 5 + Avicel PH-1010 (10% b.w.)	0	23.8	1.6
		0.5	20.7	1.7

**Table 9:** Determination of Young’s modulus for selected composites.

Ordinal Number	Type of Composite	Magnetic Induction B (T)	Young’s Modulus E (N/m^2^)(Pa∙10^−5^)
1	Epidian 5	0	1.32
		0.5	1.16
2	Epidian 5 + Fe_3_O_4_ (10% b.w.)	0	1.47
		0.5	1.72
3	Epidian 5 + Avicel PH-1010 (10% b.w.)	0	1.49
		0.5	1.22

**Table 10:** Comparison of the mechanical bending strength of composites.

Ordinal Number	Type of Composite	Magnetic Induction B (T)	Stress during Bending σ_fM_ (MPa)	Elongation in Bending ε_fB_ (%)
1	Epidian 5	0	12.6	3.3
		0.5	14.2	2.1
2	Epidian 5 + Fe_3_O_4_ (10% b.w.)	0	30.6	1.3
		0.5	41.4	1.9
3	Epidian 5 + Avicel PH-1010 (10% b.w.)	0	50.7	1.9
		0.5	48.5	2.7

**Table 11:** Impact strength of selected composites.

Ordinal Number	Type of Composite	Magnetic Induction B (T)	Impact Strength U (kJ/m^2^)
1	Epidian 5	0	2.61
		0.5	2.94
2	Epidian 5 + Fe_3_O_4_ (10% b.w.)	0	3.68
		0.5	4.94
3	Epidian 5 + Avicel PH-1010(10% b.w.)	0	3.78
		0.5	3.82

**Table 12:** Comparison of the mechanical tensile strength of composites.

Ordinal Number	Type of Composite	Admixture Content (%)	Stress during Stretching σ_m_ (MPa)	
			B = 0 T	B = 0.5 T
1	Epidian 5 + Fe_3_O_4_	0	20.1	18.6
		10	16.2	12.9
		20	16.5	13.7
		30	18.0	15.2
2	Epidian 5 + Avicel PH-1010	0	20.1	18.6
		10	23.8	20.7
		20	24.0	21.1
		30	25.1	22.5

**Table 13:** Determination of Young’s modulus for selected composites.

Ordinal Number	Type of Composite	Admixture Content (%)	Young’s Modulus E (N/m^2^)(Pa∙10^−5^)	
			B = 0 T	B = 0.5 T
1	Epidian 5 + Fe_3_O_4_	0	1.32	1.16
		10	1.47	1.72
		20	2.17	2.44
		30	1.50	1.81
2	Epidian 5 + Avicel PH-1010	0	1.32	1.16
		10	1.49	1.22
		20	1.79	1.53
		30	1.48	1.20

**Table 14:** Comparison of the mechanical bending strength of composites.

Ordinal Number	Type of Composite	Admixture Content (%)	Stress during Bending σfM (MPa)	
			B = 0 T	B = 0.5 T
1	Epidian 5 + Fe3O4	0	12.6	14.2
		10	30.6	41.4
		20	48.4	55.3
		30	41.4	49.1
2	Epidian 5 + Avicel PH-1010	0	12.6	14.2
		10	50.7	48.5
		20	22.9	21.6
		30	16.8	16.1

**Table 15:** Impact strength of selected composites.

Ordinal Number	Type of Composite	Admixture Content (%)	Impact Strength U (kJ/m^2^)	
			B = 0 T	B = 0.5 T
1	Epidian 5 + Fe_3_O_4_	0	2.61	2.94
		10	3.68	4.94
		20	4.01	5.46
		30	2.84	3.75
2	Epidian 5 + Avicel PH-1010	0	2.61	2.94
		10	3.78	3.82
		20	7.35	8.05
		30	3.80	4.08

**Table 16:** Absorptivity of composite.

Ordinal Number	Type of Composite	Admixture Content (%)	Absorptivity (%)	
			B = 0 T	B = 0.5 T
1	Epidian 5 + Fe_3_O_4_	0	0.4083	0.4189
		10	0.2491	0.2328
		20	0.3012	0.3057
		30	0.2488	0.2523
2	Epidian 5 + Avicel PH-1010	0	0.4083	0.4189
		10	0.3769	0.2786
		20	0.5027	0.4170
		30	0.8927	0.5238

**Table 17:** Frost resistance of composites.

Ordinal Number	Type of Composite	Admixture Content (%)	Frost Resistance (%)	
			B = 0 T	B = 0.5 T
1	Epidian 5 + Fe_3_O_4_	0	−0.0408	−0.5363
		10	−0.0464	0.9349
		20	−0.0305	−1.0590
		30	−0.0480	6.0255
2	Epidian 5 + Avicel PH-1010	0	−0.0408	−0.5363
		10	−0.0716	−0.4740
		20	−0.1025	−0.9349
		30	−0.2160	−5.1313

**Table 18:** Chemical resistance to acid (H_2_SO_4_).

Ordinal Number	Type of Composite	Admixture Content (%)	Chemical Resistance (%)	
			B = 0 T	B = 0.5 T
1	Epidian 5 + Fe_3_O_4_	0	0.0378	0.5092
		10	0.1704	0.0865
		20	0.1300	0.0791
		30	0.0295	0.0208
2	Epidian 5 + Avicel PH-1010	0	0.0378	0.5092
		10	0.0432	0.0196
		20	0.1135	0.0197
		30	0.0039	0.0274

**Table 19:** Chemical resistance to alkali (NaOH).

Ordinal Number	Type of Composite	Admixture Content (%)	Chemical Resistance (%)	
			B = 0 T	B = 0.5 T
1	Epidian 5 + Fe_3_O_4_	0	−0.2714	0.3830
		10	−0.4050	0.0059
		20	−0.4192	0.0473
		30	−0.3748	−0.0156
2	Epidian 5 + Avicel PH-1010	0	−0.2714	0.3830
		10	−0.2815	−0.0071
		20	−0.3283	−0.0129
		30	−0.3500	0.0343

## Data Availability

Data is contained within the article.
